# Grocery Delivery to Support Healthy Weight Gain Among Pregnant Young Women With Low Income: Protocol for a Randomized Controlled Trial

**DOI:** 10.2196/40568

**Published:** 2022-08-05

**Authors:** Marika Waselewski, Melissa Plegue, Kendrin Sonneville, Ken Resnicow, Aisha Ghumman, Cara Ebbeling, Elham Mahmoudi, Ananda Sen, Julia A Wolfson, Tammy Chang

**Affiliations:** 1 Department of Family Medicine University of Michigan Ann Arbor, MI United States; 2 School of Public Health University of Michigan Ann Arbor, MI United States; 3 New Balance Foundation Obesity Prevention Center Boston Children’s Hospital Boston, MA United States; 4 Department of Pediatrics Harvard Medical School Boston, MI United States; 5 Institute for Healthcare Policy and Innovation University of Michigan Ann Arbor, MI United States; 6 Department of Biostatistics University of Michigan Ann Arbor, MI United States; 7 Bloomberg School of Public Health Johns Hopkins Baltimore, MD United States

**Keywords:** pregnancy, weight, diet, grocery delivery

## Abstract

**Background:**

Excessive weight gain during pregnancy is associated with complications for both the mother and her infant including gestational diabetes, hypertensive disorders, operative delivery, and long-term obesity. A healthy diet during pregnancy promotes healthy gestational weight gain and determines fetal epigenetic programming in infants that impacts risk for future chronic disease.

**Objective:**

This project will examine the impact of grocery delivery during pregnancy on the weight, diet, and health outcomes of young pregnant women and their infants.

**Methods:**

A three-arm randomized controlled trial design will be performed. A total of 855 young pregnant women, aged 14-24 years, from across the state of Michigan will be enrolled and randomized equally into the three study arms. Participants in arm one (control) will receive usual care from the Special Supplemental Nutrition Program for Women, Infants, and Children (WIC); arm two will receive WIC plus biweekly grocery delivery; and arm three will receive WIC plus biweekly grocery and unsweetened beverage delivery. Weight will be assessed weekly during pregnancy, and total pregnancy weight gain will be categorized as above, below, or within guidelines. Additionally, dietary intake will be assessed at three time points (baseline, second trimester, and third trimester), and pregnancy outcomes will be extracted from medical records. The appropriateness of pregnancy weight gain, diet quality, and occurrence of poor outcomes will be compared between groups using standard practices for multinomial regression and confounder adjustment.

**Results:**

This study was funded in April 2021, data collection started in December 2021, and data collection is expected to be concluded in 2026.

**Conclusions:**

This study will test whether grocery delivery of healthy foods improves weight, diet, and pregnancy outcomes of young moms with low income. The findings will inform policies and practices that promote a healthy diet during pregnancy, which has multigenerational impacts on health.

**Trial Registration:**

ClinicalTrials.gov NCT05000645; https://clinicaltrials.gov/ct2/show/NCT05000645

**International Registered Report Identifier (IRRID):**

DERR1-10.2196/40568

## Introduction

The United States has one of the highest adolescent pregnancy rates among high-income countries with almost 800,000 15- to 24-year-old women giving birth in 2021 [1-3]. Among youth who give birth, over half gain excessive weight during pregnancy [4-8]. Excessive weight gain during pregnancy is associated with complications for both the mother and her infant including gestational diabetes, hypertensive disorders, operative delivery, and long-term obesity [5,9-15]. Young women with low income and those from racial/ethnic minority groups have the highest rates of adolescent pregnancy and face significant barriers to healthy food and beverage consumption and physical activity during pregnancy [16-21].

A healthy diet during pregnancy promotes healthy weight gain among mothers [22] and impacts permanent fetal epigenetic programming that determines future risk for chronic disease among infants [23,24]. However, most youth consume suboptimal diets that can lead to unhealthy weight gain in pregnancy [25-28]. Sugar-sweetened beverage (SSB) consumption during pregnancy is further associated with lower diet quality and greater total energy intake among pregnant women [29].

Grocery delivery is a well-established and inexpensive service that removes logistical barriers to obtaining healthy foods and beverages. There are numerous grocery delivery services (eg, Shipt.com, Instacart, FreshDirect, Amazon Fresh, Peapod, and Google Express) that help make obtaining fresh food and groceries more convenient for those who cannot or do not shop in person. However, grocery delivery is predominantly used by affluent middle-aged women [30,31], and few efforts have yet explored online grocery ordering and delivery for young pregnant Special Supplemental Nutrition Program for Women, Infants, and Children (WIC) beneficiaries [32], a population with significant logistical barriers to obtaining healthy foods.

To address this critical problem, we developed Special Delivery, a program that uses grocery delivery to overcome these logistical barriers to a healthy diet during pregnancy among pregnant young women with low income to prevent excessive gestational weight gain.

The aim of this randomized controlled trial is to assess whether grocery delivery of healthy foods to the homes of young women during pregnancy will promote healthy pregnancy weight gain. The study will also assess secondary outcomes of diet quality, pregnancy complications, and delivery outcomes. Our long-term goal is to identify effective interventions to support a healthy diet during pregnancy and promote healthy weight gain among pregnant young women with low income.

The primary hypotheses to be tested are that the home delivery of healthy foods will promote healthy weight gain during pregnancy and that the addition of unsweetened beverage delivery will promote more healthy weight gain. The findings will inform policies and practices that promote a healthy diet during pregnancy, which may have multigenerational impacts.

## Methods

### Study Design

**Figure 1:**
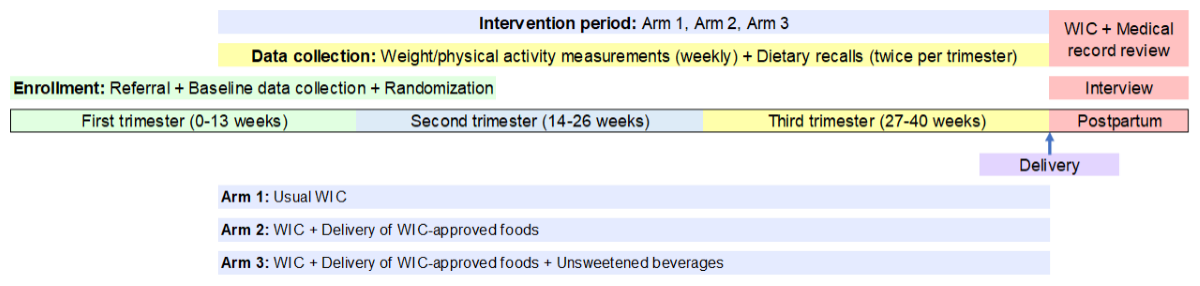
Participant study timeline. WIC: Special Supplemental Nutrition Program for Women, Infants, and Children.

This study is a three-arm randomized controlled trial with an additive parallel design. Enrollment in the study is rolling and starts as early as possible in the pregnancy (by 21 weeks’ gestation). The intervention period begins at enrollment and continues to the end of pregnancy/birth (Figure 1). Data collection occurs throughout the study with surveys at baseline, the second trimester, the third trimester, and after delivery, as well as short weekly assessments. Upon enrollment in the study, participants will be randomized to one of three groups: usual WIC (arm 1; control), usual WIC + delivery of WIC-approved food (arm 2), and usual WIC + delivery of WIC-approved food plus unsweetened beverages (arm 3).

Specifically, we will be testing the following hypotheses:

- Mothers who receive free grocery delivery of WIC-approved healthy foods will have healthier weight gain during pregnancy, higher-quality diets, and fewer perinatal complications than peers with usual WIC benefits
- Mothers who receive free grocery delivery of WIC-approved healthy foods plus unsweetened beverages will have healthier weight gain during pregnancy, higher-quality diets, and fewer perinatal complications than peers with usual WIC benefits and home delivery of WIC-approved foods only

The rationale for the study is that grocery delivery will address barriers to accessing healthy foods and will therefore facilitate a healthier diet and prevent excessive weight gain during pregnancy.

### Ethics Approval

This study was approved by the University of Michigan Institutional Review Board (HUM00190614).

### Participants

The study will include 855 young moms who are pregnant with their first child. Those with previous births are excluded to limit the impact of variable past pregnancy experiences and habits on the intervention. Participants will be included from across the state of Michigan based on the criteria below.

- Inclusion criteria
  Youth (ages 14-24 years)
  Enrolled in WIC
  Gestational age ≤20 weeks
  SMS text message capability
  Healthy singleton pregnancy
  Nulliparous
  Consume SSBs
  Living within delivery zone of a grocery delivery service
- Exclusion criteria
  Non-English speaking
  Participants who live at the same address
  High-risk pregnancy requiring specialized care

### Procedure

Participants in this study will be referred from WIC offices, online study recruitment sites, or other clinical and community organizations that serve young pregnant women across the state of Michigan. Interested women complete an online screening and enrollment survey or call the study line for assistance in completing the survey. Consent is obtained verbally or in completion of the online enrollment form and a copy of the consent form is emailed to participants.

### Sample Size Determination

Based on nationally representative studies of weight gain in pregnancy [4], we anticipate 30%, 45%, and 60% of participants in arm 1 (control), arm 2 (WIC + food delivery), and arm 3 (WIC + food plus unsweetened beverage delivery), respectively, to gain weight within the Institute of Medicine guidelines [5,33]. The sample size was determined using methods for comparing independent proportions, and a conservative alpha value of .01 was set for significance to address the multiple testing of 3 comparisons. A sample size of 774 (258 per arm) after attrition will have over 80% power (2-tailed) to detect these differences as statistically significant. Accounting for an anticipated 10% attrition rate based on our preliminary study and low-burden design, we will aim to recruit 285 participants per study arm (855 total).

### Randomization

Upon completion of baseline screening and assessments, 855 pregnant young women will be randomly assigned to either the control group or one of the two experimental groups. We will use county-level block randomization to assign participants in a 1:1:1 ratio to the three study arms. The treatment assignments will be created and stored in the Consulting for Statistics, Computing and Analytics Research randomization tool managed by the University of Michigan. No treatment assignments will be made until after collection of baseline data (consent, demographic survey, diet recalls, and other baseline questionnaires). At this time, the participant will be notified of their arm assignment and additional details required for grocery delivery (delivery address, day, and time) will be collected from participants in arms 2 and 3. All personnel who assess study outcomes are masked to group assignment. Participants and staff implementing the grocery delivery are not masked to group assignment.

### Intervention

All groups will receive usual WIC nutritional assessment and counseling benefits, including monthly nutritional counseling sessions based on a state-approved curriculum with trained nutritionists and peer counselors [34,35]. These sessions are designed to support each woman’s motivations for healthy diet in pregnancy and include education on healthy diets, portion sizes, minimizing SSB intake, cooking skills, and recipes. The intervention period will begin at study enrollment and continues until the end of pregnancy (termination or miscarriage) or infant birth.

The control group (arm 1) will receive usual WIC counseling, as described above, and food benefits loaded onto their Electronic Benefits Transfer (EBT) card to use in person at approved grocery stores. At completion of study participation, arm 1 participants will receive a 1-year prepaid subscription to a grocery delivery service of their choosing.

Participants in arm 2 (WIC counseling + food delivery) will choose WIC-approved foods from an online survey, which will be purchased and delivered to their home biweekly by the study team via a grocery delivery service at no cost to the participant. Fruit and vegetable options will be offered as well as whole grains (cereal, bread, pasta) and proteins (milk, egg, cheese) as listed in the WIC-approved foods booklet (Textbox 1) [36]. The quantities of food will follow the WIC-approved allocations for each month. Participants must text or call the study team to confirm they received the food. These deliveries are not meant to replace their normal grocery shopping or use of WIC benefits but rather to make healthy eating more convenient, and participants are not restricted in their use of WIC food benefits by participating in this program. These foods are in addition to the WIC benefits they already receive.

**Example grocery delivery options.**

**Vegetables**

Baby carrots, broccoli, bell peppers, cauliflower, radishes, snap peas, green peas, avocados, tomatoes, cucumbers, celery, mushrooms, spinach, corn.

**Fruits**

Apples, pears, oranges, clementines, grapes, bananas, nectarines, peaches, strawberries, blueberries, raspberries, blackberries, watermelon, pineapple.

**Proteins and grains**

Yogurt, cheese, milk, whole grain bread, whole grain buns, whole grain tortillas, brown rice, whole grain cereal, beans, lentils, peanut butter, eggs.

**Unsweetened beverages**

Seltzer water, unsweetened tea, Spindrift, sparkling tea, bottled water

Participants in arm 3 (WIC counseling + food plus unsweetened beverage delivery), the home delivery of healthy foods, as described above, will be supplemented with the inclusion of participant-selected unsweetened beverages, in quantities comparable to their current SSB intake (based on self-reported consumption). Unsweetened beverages are intended to replace current SSB consumption. Because SSBs contribute to a significant number of excess calories among youth, this group includes unsweetened beverage delivery to further promote healthy weight gain. The unsweetened beverage choices will include bottled water (flavored or unflavored), unsweetened seltzer water (flavored or unflavored), and unsweetened teas. Artificially sweetened beverages and SSBs will be excluded.

### Participant Retention

Our study is designed to be low burden, so participants only have to maintain minimal communication via text, phone call, or email to remain in the study. In addition, participants are incentivized with Amazon gift cards for various components of the study. Participants in all arms receive US $20 for completing the enrollment process, baseline survey, first diet recall, and medical record request form. Participants in all arms will receive US $20 for each additional diet survey completed on their own throughout the course of the study (up to 5 total). For each week participants use their study provided scale, they will receive US $1, and an additional US $10 is given for the end-of-study interview. Women in the intervention arms receive biweekly grocery deliveries during their pregnancy, while women in the control arm receive free access to 1 year of a grocery delivery service at the end of their pregnancy. The resulting maximum incentive for participation is US $160 for participants who enroll at 10 weeks’ gestation, complete all study questionnaires, and remain active for the remaining 7 months of their pregnancy.

### Data Collection

At baseline, interested and eligible participants will complete the consent process to participate and respond to baseline measures, described in detail below. In addition to the baseline surveys, participants will be asked to share weight, physical activity, and diet recall data at specified times throughout the study period. The complete list of outcomes, covariates, and timeline of assessments is shown in Table 1.

**Table 1:** Study outcomes and covariates with timeline of data collection.

Measure			Data source		Time points
**Primary outcome**					
	Total and weekly weight gain in pregnancy: above, below, or within guidelines	Prepregnancy weight: self-report and medical record review Pregnancy weight gain: BodyTrace scale and medical record review		At enrollment, weekly during pregnancy	
**Secondary outcomes**					
	Diet quality: Healthy Eating Index score	Paired ASA24^a^ diet recall surveys		Twice at baseline, twice at second trimester, twice third trimester	
	Prenatal complications: gestational diabetes, hypertensive disorders; delivery complications: operative delivery, shoulder dystocia, postpartum hemorrhage; and birth weight: small/large for gestational age	Medical record review		Postpartum	
**Process measures**					
	Delivery of WIC^b^-approved food and unsweetened beverages	Online grocery delivery orders; requested monthly by participants		Biweekly deliveries	
	Experience with deliveries	Phone interview		Postpartum	
	Medical/WIC record release	SignNow e-signature		At enrollment	
**Confounders/modifiers**					
	Age, race, ethnicity, socioeconomic status, home environment, food insecurity	Demographic survey		At enrollment	
	Physical activity	Physical activity survey		Weekly during pregnancy	
	Food behaviors	Cooking frequency, fast-food frequency, cooking confidence, eating disorder examination		At enrollment, postpartum	
	Body shape preference	Pulvers body image instrument		At enrollment, postpartum	
	WIC nutrition visits and food benefits redeemed	WIC record review		Postpartum	

^a^ASA24: Automated Self-Administered 24-hour.

^b^WIC: Special Supplemental Nutrition Program for Women, Infants, and Children.

### Sociodemographic Data

All sociodemographic data will be collected upon enrollment online or by phone with the study team. These include date of birth (to calculate age), self-reported race and ethnicity, highest level of education, free/reduced lunch status while in school (a valid measure of socioeconomic status among youth) [37], full address including zip code (for grocery delivery and delivery of BodyTrace scale), home environment (validated Home Food Environment assessment [38,39], other individuals in household, access to transportation), and a validated two-question food insecurity screen for youth to determine baseline access to food [40,41]. Participants’ and household members’ food allergies and cultural/religious food preferences will also be collected upon enrollment.

### Weight and BMI

Prepregnancy weight and height will be obtained by self-report and verified by medical record review after birth to determine prepregnancy BMI and identify target weight gain zones. Weekly weight measurements during pregnancy will be measured via a BodyTrace scale and recorded automatically using cellular connectivity (no Wi-Fi or cellular plan required) [42]. The scale will be mailed to participants’ homes upon enrollment. The BodyTrace scale provides valid weight measures and has been used by several other large-scale weight interventions [43-45].

### Dietary Intake

Dietary intake will be measured with paired assessments using the Automated Self-Administered 24-hour (ASA24) dietary recall survey [46-48]. This process has been validated by comparison to in-person 24-hour recalls and has been used by other studies of pregnant youth with low income [49,50]. At enrollment, participants will complete their first recall over the phone with the study team. A second baseline ASA24 survey will then be completed, approximately 2 to 5 days after enrollment. Participants will complete four additional ASA24 surveys using the same paired system during the intervention period. Two will be completed in the second trimester and two in the third trimester. Study team members will be available, as needed, to assist participants in completing all ASA24 surveys. Paired responses will be combined and used to calculate a Healthy Eating Index (HEI) score for each time point: baseline, second trimester, and third trimester [46,51]. The output from the ASA24 includes additional data on fruit and vegetable servings as well as other macronutrient data.

### Physical Activity

The American College of Obstetricians and Gynecologists recommends an exercise program of moderate intensity exercise for at least 20 to 30 minutes per day on most or all days of the week for pregnant women [52]. To measure physical activity, we will use an adaptation of the Youth Risk Behavior Survey and National Youth Physical Activity and Nutrition Study survey item that has been validated with accelerometer data [53-56]. The survey will be sent weekly via SMS text message to assess the proportion of time youth meet the guidelines, as physical activity impacts weight gain in pregnancy.

### Body Size Preference

Participants will complete the validated Pulvers Body Image [57] scale at enrollment and during the phone interview after birth to assess their body size preferences. At baseline, participants will be asked to identify their prepregnancy body shape and ideal body shape from the 9 options included in the Pulvers scale. At follow-up, participants will be asked to identify their current (postpartum) body shape and ideal body shape. Participants’ preference in body size may impact their weight-related behaviors and provides critical context on whether they wish to be or stay bigger after pregnancy or try to return to their prepregnancy size [58].

### Food Behaviors

Participants will be prompted to complete questions adapted from the Eating Disorder Examination Questionnaire Short and Youth Eating Disorder Examination-Questionnaire at baseline and end of study to identify perceptions of loss of control of eating, binge eating, weight/shape overvaluation, and body dissatisfaction [59-61]. Additional questions drawn and adapted from the National Health and Nutrition Examination Survey focused on the frequency of home-cooked meals in the household and fast-food meals [62] will also be asked as well as participants’ confidence in cooking meals [63,64]. These questions will be assessed during the baseline and end-of-study survey assessments.

### Text Message Reminders

SMS text messages will be sent to participants in all arms using Textizen.com, a GovDelivery platform. Participants will receive 2 to 3 SMS text messages per week reminding them to confirm their delivery, place their order, step on their scale, and respond to physical activity questions. These SMS text messages will also prompt participants to share any updates they may have regarding their pregnancy, allowing participants an opportunity to report on infant birth, miscarriage, or any other outcomes. In addition, participants will be prompted via SMS text message to complete their diet recall surveys throughout their pregnancy.

### Medical Records Data

Medical records will be requested from participants’ prenatal and delivery care sites within a few weeks of delivery using the release forms completed at enrollment. Data collected will include all health care visits during pregnancy and up to 12 weeks postpartum, including prenatal visits (diagnoses and complications), nutrition visits (number and content of visits), emergency department visits, inpatient stays, WIC counseling visits (number and type: nutrition vs breastfeeding), quantity of WIC foods redeemed, laboratory results (1-hour glucose tolerance test, A1C, blood glucose levels, complete blood count, urine studies, liver/renal studies), vital signs (weight, height, blood pressure, pulse), medications prescribed, complications during prenatal care (gestational diabetes, hypertensive disorders), infant birth weight, and gestational age at delivery.

### End-of-Study Interview

A phone interview will be performed after each woman gives birth to collect qualitative insights into participants’ experience with grocery/beverage delivery; the impact of delivery on their diet; overall WIC experiences; and end-of-pregnancy body preference, self-reported weight gain, physical activity, any prenatal or childbirth complications, plans for breastfeeding, and food insecurity.

### Data Analysis

To understand the effect of grocery delivery on pregnancy weight gain, diet quality, and health outcomes, weight will be measured weekly, dietary quality will be assessed twice per trimester, and perinatal outcomes will be assessed at the end of the pregnancy. All analyses will follow the intention-to-treat principle. Prior to analysis, assessment of missing data will be completed and appropriately imputed using multiple imputation methods [65].

Our primary outcome is appropriate weight gain in pregnancy, which is based on prepregnancy BMI and includes weekly weight gain and total weight gain recommendations. Total weight gain at birth will be categorized as above, below, or within the Institute of Medicine guidelines [5,33]. Multinomial logistic regression analysis will be used to compare the likelihood of falling above, within, or below guidelines for total weight gain in pregnancy across arms after adjusting for potential confounders such as age, race, ethnicity, socioeconomic status, and household characteristics. Confounders will be identified through significant findings in bivariate analysis comparing the three groups and by consideration of variables based on prior scientific evidence. Summary statistics of key time-dependent measures and other non–time-dependent measures will also be included as covariates. Modification of the study arm effect by key variables will be investigated through interaction terms in the regression model.

The trajectory of weight gain behavior, using each week’s categorized weight gain (within guidelines vs not), will additionally be assessed as the outcome in a clustered logistic regression model fit using a generalized linear mixed model framework. The study arm is the primary between-participant factor, while time (week) is the main within-participant covariate. The interaction term between time and arm is also a factor of interest, as it will allow us to assess changes in weight gain behaviors over time between study arms. A random participant-level intercept will account for within-participant clustering, and a random slope for time will be investigated to account for additional between-participant variability. Additional covariates and effect modifiers included in these regression models will be chosen using the same methods as before.

Our secondary outcome is diet quality as measured by the HEI score based on two ASA24 measurements at baseline and in the second and third trimesters [51,66]. A comparison of overall HEI scores between study arms will be carried out using separate linear mixed models with calculated HEI scores from each trimester as the outcome. As before, the study arm will be the primary between-participant factor; time, as measured by trimester, the within-participant covariate; and the interaction between arm and time will be assessed to measure the difference in trimester effect between the arms. Random participant-level intercepts and slopes for time will assess the deviation from the overall average and account for any within-participant clustering. Models will be adjusted for additional confounders and covariates, determined as in prior models. Fruit and vegetable intake, as well as other macronutrient details, will also be evaluated.

Prenatal complications, delivery complications, and birth weight (small/large for gestational age) are also secondary outcomes that will be assessed. Comparison of the proportion of participants with complications between study arms will be analyzed using a logistic regression model in a manner analogous to the primary outcome of appropriate weight gain in pregnancy.

The end-of-study interview transcripts will be analyzed using standard qualitative techniques [67-69]. Two investigators will review all transcripts, create memos of major concepts present, and iteratively develop a codebook. Transcripts will be coded and major themes and conclusions determined by consensus. Themes identified from these transcripts will describe participants’ satisfaction or dissatisfaction with delivery, perceived impact of food delivery on their health and diet, and other challenges/barriers to healthy behaviors during pregnancy. The interviews will be used to provide context and narrative to inform findings from quantitative outcome data, and will be used in dissemination to tell the participants’ perspective regarding the impact of grocery delivery.

Finally, we will conduct a cost analysis focused on costs from the payer perspective during the pregnancy period. All costs will be adjusted to current dollars and will represent average cost per participant. Our analysis will estimate the costs of delivering the intervention, including cost of grocery delivery service, increased costs for WIC due to increased redemption of food benefits [70], print and online media advertisements to maximize uptake, costs to train WIC staff on new procedures, and increased WIC visit time to allow staff to educate beneficiaries. Medical records data will also be used to identify the number and type of all medical care used by participants during pregnancy, including prenatal care visits, specialist visits and consultations, emergency department visits, inpatient care, and delivery care. The average total health care costs per participant associated with each arm of the trial will be calculated using nationally representative health care expenditure data from the most recent Medical Expenditure Panel Survey [71]. Although medical records data from their usual sources of care may not include visits to urgent care and outside facilities, this will not likely introduce bias due to randomization to study arms.

## Results

This study was funded in April 2021, data collection started in December 2021, and data collection is expected to be concluded in 2026. No results are available as of manuscript preparation.

## Discussion

This protocol describes a randomized controlled trial to evaluate whether grocery delivery of healthy WIC-approved foods supports healthy pregnancy weight gain for young pregnant women with low income. We will additionally evaluate if the grocery delivery impacts diet quality, pregnancy complications, and delivery outcomes. We anticipate that the young pregnant women in the two intervention groups will have better rates of appropriate pregnancy weight gain, improved diet quality, and lower rates of pregnancy and delivery complications.

Results from this study can be used to inform United States Department of Agriculture policies on online use of WIC benefits as well as to provide evidence about the cost-effectiveness of national implementation of grocery delivery for WIC beneficiaries [32]. These outcomes may additionally provide evidence to clinical practices or health systems on opportunities for the use of food delivery programs within their patient populations to address social determinants of health.

There are several key strengths to this protocol. This work uses a low-cost well-established service to enhance an existing government program which enables the results to inform policies to improve the implementation of WIC services and other programs for vulnerable populations. COVID-19 has also led to a surge in online grocery ordering, which enhances the generalizability of this study [31]. Additionally, this study uses low-burden communication and data collection methods to allow for high levels of participant retention.

Limitations of this study are that it does not perfectly emulate the real-life application of integrating grocery delivery into WIC services, as study personnel are ordering and managing the delivery process, which reduces the generalizability of the results to program-based implementation. Specifically, participants request the groceries from the study team who then place the orders and manage the deliveries. Additionally, participants are able to access and use their WIC food benefits in addition to groceries provided, and it does not directly replace this benefit.

Ultimately, this study will provide rigorous evidence regarding the impact of grocery delivery of healthy foods and unsweetened beverages among pregnant young women with low income and their infants. The findings from this study will provide invaluable information about the potential benefits of incorporating grocery delivery in WIC and about the costs to implement, sustain, and expand such a program.

## Appendix

Multimedia Appendix 1 Peer Review Report from the Community-Level Health Promotion (CLHP) Study Section - Healthcare Delivery and Methodologies Integrated Review Group - Center For Scientific Review (National Institutes of Health, USA).

## Abbreviations

ASA24: Automated Self-Administered 24-hour
HEI: Healthy Eating Index
SSB: sugar-sweetened beverage
WIC: Special Supplemental Nutrition Program for Women, Infants, and Children
