# Supplementary material for: Grocery Delivery to Support Healthy Weight Gain Among Pregnant Young Women With Low Income: Protocol for a Randomized Controlled Trial
Source: JMIR Res Protoc. 2022 Aug 5;11(8):e40568. doi: 10.2196/40568 (PMC9391971; doi:10.2196/40568)
Supplement: Multimedia Appendix 1 [file resprot_v11i8e40568_app1.pdf]

SUMMARY STATEMENT
(Privileged Communication)

PROGRAM CONTACT:
Dr. Maurice Davis
(301) 435-6887
maurice.davis@nih.gov

Release Date: 06/27/2020
Revised Date:

Application Number: 1 R01 HD101522-01A1
Formerly: 1R01HD101522-01

Principal Investigator

CHANG, TAMMY

Applicant Organization: UNIVERSITY OF MICHIGAN AT ANN ARBOR

Review Group: CLHP
Community-Level Health Promotion Study Section

Meeting Date: 06/15/2020
Council: OCT 2020
Requested Start: 12/01/2020

RFA/PA: PA18-480
PCC: PPB -MD

Project Title: Grocery Delivery to Promote Healthy Weight Gain Among Low-Income Pregnant Young Women
SRG Action: Impact Score:23 Percentile:3
Next Steps: Visit https://grants.nih.gov/grants/next_steps.htm
Human Subjects: 48-At time of award, restrictions will apply
Animal Subjects: 10-No live vertebrate animals involved for competing appl.
Gender: 2A-Only women, scientifically acceptable
Minority: 1A-Minorities and non-minorities, scientifically acceptable
Age: 7A-Only Adults, scientifically acceptable

Project Year	Direct Costs Requested	Estimated Total Cost
1	499,999	779,998
2	499,999	779,998
3	499,999	779,998
4	499,999	779,998
5	499,999	779,998
TOTAL	2,499,995	3,899,990

ADMINISTRATIVE BUDGET NOTE: The budget shown is the requested budget and has not been adjusted to reflect any recommendations made by reviewers. If an award is planned, the costs will be calculated by Institute grants management staff based on the recommendations outlined below in the COMMITTEE BUDGET RECOMMENDATIONS section.
EARLY STAGE INVESTIGATOR
NEW INVESTIGATOR

1R01HD101522-01A1 Chang, Tammy

EARLY STAGE INVESTIGATOR
NEW INVESTIGATOR
PROTECTION OF HUMAN SUBJECTS UNACCEPTABLE

RESUME AND SUMMARY OF DISCUSSION: This study aims to evaluate the impact of grocery delivery during pregnancy on weight gain and diet among low-income pregnant young women with limited access to healthy foods. The focus of the study on the access to healthy foods to prevent excessive weight gain during pregnancy is very significant. The investigative team assembled by this new investigator is multidisciplinary and has a good relationship with the WIC program. The research environment is excellent with strong letters of support. The revised application is responsive to most of the critiques from prior review. Other strengths include the use of RCT design, the high potential scalability, the team's experience in text messaging, the improved measures of diet, the adequate sample size, and the appropriate analytic plan. While the home environment measures have been added, some reviewers felt that how the home environment might contribute to weight gain needs to be further discussed. Some reviewers felt that the juice in the WIC food package needs to be better addressed, while others did not think it was an issue. More attention should also be paid to the effect of the SNAP participation. Overall, however, the Committee agreed that identified weaknesses are minor. This is a strong application from an excellent research team, and the enthusiasm was high.

DESCRIPTION (provided by applicant): Excess weight gain during pregnancy among low income pregnant youth is associated with serious multi- generational morbidity for both the mother and infant. Healthy diet and limiting sugar sweetened beverages during pregnancy promotes healthy weight gain during pregnancy and impacts permanent fetal genetic programming that determines risk for chronic disease among infants. Making healthy diet choices convenient improves quality of diet among youth. The Special Supplemental Nutrition Program for Women, Infants, and Children (WIC) benefits cover the cost of healthy foods for low income pregnant women, but most young mothers are not able to obtain the food due to logistical barriers such as transportation. Grocery delivery is a well-established and inexpensive service that removes logistical barriers to obtaining healthy food but is underused by those that may need it most -young pregnant women. The objective of this application is to determine the impact of delivering healthy foods during pregnancy on the health of low-income young women and their infants. For this three-armed randomized control trial, subjects are pregnant young women ≤24 years of age who are giving birth for the first time and enrolled in WIC (Arm 1: Usual WIC (Control), Arm 2: Delivery of WIC-approved food only, Arm 3: Delivery of WIC-approved food PLUS unsweetened beverages). Subjects are randomly assigned to experimental or control groups. Weight and dietary data will be recorded throughout their pregnancy for all groups. In both experimental groups (Arm 2 & 3), the intervention will last from the first trimester of their pregnancy to the birth of their infant. Arm 2 will receive healthy food delivery every two weeks. Arm 3 will receive healthy food and unsweetened beverage delivery every two weeks. This project will advance the scientific knowledge about the effect of a simple and inexpensive intervention (grocery delivery) on weight gain and dietary behaviors among low-income pregnant young women, which can be applied to policies and practices that affect other vulnerable populations.

PUBLIC HEALTH RELEVANCE: Excessive weight gain during pregnancy among pregnant young women is associated with serious multi- generational morbidity for both the mother and infant. This project will advance the scientific knowledge about the effect of grocery delivery on weight gain and diet during pregnancy among low-income pregnant young women who have limited access to healthy foods.

CRITIQUE 1

Significance: 3
Investigator(s): 2
Innovation: 3
Approach: 4
Environment: 1

Overall Impact: This is a resubmission application from an accomplished junior investigator that will examine the effect of grocery delivery during pregnancy on diet and weight gain in low-income pregnant young women with overweight or obesity. It is a 3 arm study: usual WIC care (Arm 1), home delivery of WIC-approved foods only (Arm 2), or Arm 2 combined with unsweetened beverages (Arm 3). Primary outcome is gestational weight gain (total gestational weight gain and weekly weight gain assessed using bodytrace 3G scales); secondary outcomes include quality of diet (assessed using the ASA24, calculating the healthy eating index score), pregnancy outcomes (complications, birth weight), process outcomes (experience with food deliveries, adequate delivery of items), and cost. The proposal addresses an important public health issue as excessive weight gain during pregnancy is an important determinant of pregnancy and infant outcomes in the targeted population. The multidisciplinary team is strong with requisite expertise to successfully accomplish the stated aims. The potential scalability of the intervention is a notable strength. The team responded to several of the concerns noted by reviewers in the first submission; improved their assessment of diet, discussed that WIC will be providing instructions and training on food preparation (albeit without additional training from the research team), improved their assessment of the home food environment (to better assess this as a potential confounder), and discussed their focus on youth ages 14-24 years (as these are most at-risk for logistical barriers for obtaining healthy foods due to limited transportation). Despite these modifications, the team did not address some of the critical limitations noted in the first application, particularly with regard to 1) solely addressing issues of access to drive gestational weight gain, when healthy eating and weight gain is due to a multitude of factors as described in the 2009 IOM conceptional model of determinants/guidelines (and data from the PIs own qualitative study revealed that convenience was only one factor influencing mother's gestational weight gain; however, stress was another significant factor but not being addressed in this proposal); 2) evidence that having WIC available foods is associated with excessive gestational weight gain (while WIC may improve mothers' nutrition, limited to no data indicate the WIC food package decreases the consumption of unhealthy foods bought by other members in the home or from the corner store, or affects stress and motivation, factors that may be important for young moms who live in multigenerational households and don't have control over the food environment). Overall, however, impact is thought to be high.

1. Significance

Strengths

- Excess gestational weight gain is an important determinant of pregnancy and postpartum outcomes for mom and baby.
- Sugar sweetened beverages are a large source of calories and contribute to excessive gestational weight gain; interventions to address SSBs may reduce gestational weight gain.
- Few interventions are focused on adolescents and young adults.
- Grocery delivery may reduce barriers to healthy food access.

Weaknesses

- Access is clearly not the only variable impacting nutrition and healthy weight among teens and young adults.

- No mention of the fact that the WIC food package encourages the consumption of juice and includes this as part of their benefit package (and how this may influence weight).

2. Investigator(s)

Strengths

- Key personnel represent a range of experience related to the project including family medicine (Chang), nutritional and behavioral intervention research (Resnicow, Sonneville), cost effectiveness (Mahmoudi), health policy (Wolfson), and biostatistics (Sen).

Weaknesses

- Having an investigator/key personnel from WIC would be helpful to ensure adequate buy-in by WIC staff and potential for integration/dissemination/scalability.

3. Innovation

Strengths

- Inclusion of an overlooked group – adolescents and young adults in gestational weight gain studies.

Weaknesses

- Use of bodytrace scales and text messaging is not particularly innovative.

4. Approach

Strengths

- Application builds on successful studies by Ebbeling (Consultant) and Resnicow (Co-Investigator), delivering unsweetened beverages to overweight and obese youth, along with leveraging the successful texting platform of PI.
- PI conducted a pilot study on feasibility of recruitment and acceptability of WIC grocery delivery – both were positive. Also showed in a non-randomized, small sample that there is potential to decrease rates of excessive gestational weight gain with this same approach.
- Use of ASA-24 for assessing dietary quality is much improved over a 4 item FFQ.

Weaknesses

- The team reports that WIC providers do intensive nutritional counseling – including MI – however, more evidence is needed from Michigan WIC staff to believe that busy, under-resourced WIC providers are able to do more than just adequate/cursory counseling, and counsel more than every 3 months.
- Use of self-reported pre-pregnancy weights in this population may be biased, given most of these individuals probably don’t have scales in their homes to accurately know their pre-pregnancy weights.
- While using bodytrace scales eliminates issues around transportation for assessments, connectivity can still be an issue along with user error for these to measure the primary outcome.
- While the investigators are planning to have a better self-reported inventory of the home food environment (to evaluate this variable as a potential confounder/effect modifier), their

intervention still doesn't seem to address the issues of other foods/beverages in the home and kitchen supplies, additional determinants of healthy weight gain.

- The WIC food package currently allows for juice and milk; the investigators don't discuss how they will deal with this issue (while it's 100% fruit juice and low fat milk, it still has significant quantities of sugar and many define both as a sugar sweetened beverages with deleterious effects on health, e.g., REGARDS study).

5. Environment

Strengths

- The environment and resources at University of Michigan are excellent.

Weaknesses

- None.

Study Timeline

Strengths

- Sufficient timeline provided for recruitment and intervention delivery. While enrolling more than 20 women a month seems high for this new investigator, her feasibility studies and strong team lesson concerns.

Weaknesses

- None.

Protections for Human Subjects

Acceptable Risks and/or Adequate Protections

Data and Safety Monitoring Plan (Applicable for Clinical Trials Only):

Unacceptable

- Should really have named individuals on this proposal (instead of TBD individuals).

Inclusion Plans

- Sex/Gender: Distribution justified scientifically
- Race/Ethnicity: Distribution justified scientifically
- For NIH-Defined Phase III trials, Plans for valid design and analysis: Not applicable
- Inclusion/Exclusion Based on Age: Distribution justified scientifically

Vertebrate Animals

Not Applicable (No Vertebrate Animals)

Biohazards

Not Applicable (No Biohazards)

Resubmission

- The team responded to several of the concerns noted by reviewers in the first submission; improved their assessment of diet, discussed that WIC will be providing instructions and training on food preparation (albeit without additional training from the research team), improved their assessment of the home food environment (to better assess this as a potential confounder), and discussed their focus on youth ages 14-24 years (as these are most at-risk for logistical barriers for obtaining healthy foods due to limited transportation).

Resource Sharing Plans

Not Applicable (No Relevant Resources)

Authentication of Key Biological and/or Chemical Resources

Not Applicable (No Relevant Resources)

Budget and Period of Support

Recommend as Requested

CRITIQUE 2

Significance: 2
Investigator(s): 2
Innovation: 2
Approach: 3
Environment: 2

Overall Impact: This resubmission is from a new investigator. The study aims to evaluate the impact of delivering healthy foods to WIC-enrolled pregnant women on pregnancy weight gain and dietary behaviors. It will use a RCT to compare outcomes among three groups: (1) Usual WIC (Control); (2) Delivery of WIC-approved food only; and (3) Delivery of WIC-approved food PLUS unsweetened beverages. A cost analysis from a payer's perspective will also be conducted to assist with decision-making. The study is based on prior evidence suggesting that SSB is a risk factor for overweight and is consumed by a high percentage of young pregnant women. Findings will have direct policy implications. The study is innovative focusing on a group that is often overlooked in research (pregnant young women) and using low-burden technology for the intervention and collection of data (weight). The approach is well thought out and designed with WIC collaborators and while not specifically discussed, uses basic principles of CBPR. The exclusion and inclusion criteria are appropriate; the timeline is realistic and data collection methods are feasible. The team also has prior experience working the WIC program. There are enthusiastic letters of support from WIC. Sample size is adequate. One minor concern is the potentially modifying effect of SNAP participation. WIC provides less $ for food assistance than SNAP and it is possible that SNAP participation may modify the effect. Another concern (previously noted by reviewers) is the potential for contamination.

1. Significance

Strengths

- Based on prior research showing that access to healthy foods and beverages is a barrier to healthy eating along low income pregnant women and that SSB is a risk factor for obesity and consumed by a high percentage of low income young adults.

Weaknesses

- Other risk factors were not discussed

2. Investigator(s)

Strengths

- PI is a new investigator trained in family medicine and public health with a productive record.
- Excellent team

Weaknesses

- None noted by reviewer

3. Innovation

Strengths

- Focuses on a group that is often overlooked in research (pregnant young women)
- Uses low-burden technology for the intervention and collection of data (weight)

Weaknesses

- None noted by reviewer

4. Approach

Strengths

- RCT that is feasible and well-planned.
- Intervention activities well thought out and described.
- Appropriate (and feasible) exclusion and inclusion criteria.
- Prior experience (in team) in recruitment and data collection methods.
- Process will be evaluated.
- Engagement of stakeholders including WIC collaborators in design of study (not innovative but important) and dissemination of findings.
- Prior experience working WIC.
- Sample size is adequate and analytic plan appropriate.
- Appropriate incentives for recruitment and retention of participants.

Weaknesses

- ASA24 can be tedious and it was not clear if this will be done in the WIC clinic or at home. If the latter, internet connection is required.
- SNAP participation may confound or modify effect and there was no indication that SNAP participation status would be included in the analysis (or data collection).
- Description of collection/acquisition of cost data was missing.
- There is still potential for contamination.

5. Environment

Strengths

- Excellent

Weaknesses

- None noted by reviewer

Study Timeline

Strengths

- Appropriate and feasible

Weaknesses

- None noted by reviewer

Protections for Human Subjects

Acceptable Risks and/or Adequate Protections

Data and Safety Monitoring Plan (Applicable for Clinical Trials Only):

Acceptable

Inclusion Plans

- Sex/Gender: Distribution justified scientifically
- Race/Ethnicity: Distribution justified scientifically
- For NIH-Defined Phase III trials, Plans for valid design and analysis: Not applicable
- Inclusion/Exclusion Based on Age: Distribution justified scientifically

Vertebrate Animals

Not Applicable (No Vertebrate Animals)

Biohazards

Not Applicable (No Biohazards)

Resubmission

- Previous reviewers' concerns were adequately addressed to a large extent
- There is still the potential for contamination but an attempt was made to address it.

Resource Sharing Plans

Acceptable

Authentication of Key Biological and/or Chemical Resources

Not Applicable (No Relevant Resources)

Budget and Period of Support

Recommend as Requested

CRITIQUE 3

Significance: 1
Investigator(s): 1
Innovation: 1
Approach: 2
Environment: 1

Overall Impact: This R01 resubmission seeks to conduct a 3-arm RCT to determine the impact of using twice-monthly grocery delivery services to deliver healthy foods to pregnant young (24 y or younger) primiparous WIC recipients (Arm 1: Usual WIC (Control), Arm 2: Delivery of WIC-approved food only, Arm 3: Delivery of WIC-approved food PLUS unsweetened beverages).Their overall goal is to identify effective interventions to increase the consumption of healthy foods and decrease the consumption of sugar-sweetened beverages (SSB) during pregnancy to prevent excessive weight gain among low-income pregnant young women. The significance is high, the rigor of prior research is strong, and the investigative team is strong. Level of innovation is high. The approach is feasible, and using electronic scales to objectively report the primary outcome measure is both feasible and rigorous, with high levels of fidelity. The use of participant incentives is thoughtfully and creatively considered. This project has the potential for scalability and for high impact.

1. Significance

Strengths

- Excessive gestational weight gain is an important health issue, with huge implications for the mother and future generations. Excessive weight gain is common among teen and young mothers.
- For young women receiving WIC benefits, there are often logistical barriers to obtaining the food.
- Rigor of prior research, including that done by the PI and the research team, is strong.

Weaknesses

- None noted.

2. Investigator(s)

Strengths

- Excellent expertise in behavioral interventions, social media and text messaging, family medicine, OB-Gyn, health behavior, nutritional sciences, health policy, health economics, mixed methods, and biostatistics.
- The team has a history of collaboration.

Weaknesses

- None noted.

3. Innovation

Strengths

- Grocery delivery of healthy foods is innovative.
- Text messaging to collect data is innovative.

Weaknesses

- None noted.

4. Approach

Strengths

- The Community Advisory Board is an excellent resource.
- Use of electronic scales that automatically transmit results to researchers improves fidelity of the primary outcome measure of maternal weight gain during pregnancy.
- Conducting quantitative, qualitative, and cost analyses will provide a rich, comprehensive assessment of this project.
- There is likely to be low attrition rates, given that recipients receive free groceries or have the prospect of a free grocery delivery service after baby's birth.

Weaknesses

- Conducting end-of-study qualitative interviews with all of the 855 participants may be more than is needed to reach thematic saturation.

5. Environment

Strengths

- The University of Michigan has outstanding resources.
- There are appropriate letters of support from WIC centers.

Weaknesses

- None noted.

Study Timeline

Strengths

- Appropriate.

Weaknesses

- None noted by reviewer

Protections for Human Subjects

Acceptable Risks and/or Adequate Protections

- Pregnant women and minors are both vulnerable populations, and considerations regarding this status should be outlined.

Data and Safety Monitoring Plan (Applicable for Clinical Trials Only):

Acceptable
- o acceptable

Inclusion Plans

- Sex/Gender: Distribution justified scientifically
- Race/Ethnicity: Distribution justified scientifically
- For NIH-Defined Phase III trials, Plans for valid design and analysis: Not applicable
- Inclusion/Exclusion Based on Age: Distribution justified scientifically

Vertebrate Animals
Not Applicable (No Vertebrate Animals)

Biohazards
Not Applicable (No Biohazards)

Resubmission

- This resubmission was very responsive to reviewer comments.

Resource Sharing Plans
Acceptable

Authentication of Key Biological and/or Chemical Resources
Not Applicable (No Relevant Resources)

Budget and Period of Support
Recommend as Requested

THE FOLLOWING SECTIONS WERE PREPARED BY THE SCIENTIFIC REVIEW OFFICER TO SUMMARIZE THE OUTCOME OF DISCUSSIONS OF THE REVIEW COMMITTEE, OR REVIEWERS' WRITTEN CRITIQUES, ON THE FOLLOWING ISSUES:

PROTECTION OF HUMAN SUBJECTS: UNACCEPTABLE

- o Named individuals for the Data and Safety Monitoring Board should be provided (see Critique 1).

INCLUSION OF WOMEN PLAN: ACCEPTABLE

INCLUSION OF MINORITIES PLAN: ACCEPTABLE

INCLUSION ACROSS THE LIFESPAN: ACCEPTABLE

COMMITTEE BUDGET RECOMMENDATIONS: The budget was recommended as requested.

Footnotes for 1 R01 HD101522-01A1; PI Name: Chang, Tammy

NIH has modified its policy regarding the receipt of resubmissions (amended applications).See Guide Notice NOT-OD-18-197 at https://grants.nih.gov/grants/guide/notice-files/NOT-OD-18-197.html. The impact/priority score is calculated after discussion of an application by averaging the overall scores (1-9) given by all voting reviewers on the committee and multiplying by 10. The criterion scores are submitted prior to the meeting by the individual reviewers assigned to an application, and are not discussed specifically at the review meeting or calculated into the overall impact score. Some applications also receive a percentile ranking. For details on the review process, see http://grants.nih.gov/grants/peer_review_process.htm#scoring.

MEETING ROSTER

Community-Level Health Promotion Study Section
Healthcare Delivery and Methodologies Integrated Review Group
CENTER FOR SCIENTIFIC REVIEW
CLHP
06/15/2020 - 06/16/2020

Notice of NIH Policy to All Applicants: Meeting rosters are provided for information purposes only. Applicant investigators and institutional officials must not communicate directly with study section members about an application before or after the review. Failure to observe this policy will create a serious breach of integrity in the peer review process, and may lead to actions outlined in NOT-OD-14-073 at https://grants.nih.gov/grants/guide/notice-files/NOT-OD-14-073.html and NOT-OD-15-106 at https://grants.nih.gov/grants/guide/notice-files/NOT-OD-15-106.html, including removal of the application from immediate review.

CHAIRPERSON(S)

WARD, DIANNE STANTON, EDD
PROFESSOR
DEPARTMENT OF NUTRITION
GILLINGS SCHOOL OF GLOBAL PUBLIC HEALTH
UNIVERSITY OF NORTH CAROLINA AT CHAPEL HILL
CHAPEL HILL, NC 27599

MEMBERS

ARREDONDO, ELVA M, PHD
PROFESSOR
DEPARTMENT OF HEALTH PROMOTION AND
BEHAVIORAL SCIENCE
SAN DIEGO STATE UNIVERSITY
SAN DIEGO, CA 92123

BARANOWSKI, TOM, PHD
DISTINGUISHED EMERITUS PROFESSOR
DEPARTMENT OF PEDIATRICS
CHILDREN'S NUTRITION RESEARCH CENTER
BAYLOR COLLEGE OF MEDICINE
HOUSTON, TX 77030

BERG, CARLA J, PHD
PROFESSOR
DEPARTMENT OF PREVENTION AND COMMUNITY HEALTH
GEORGE WASHINGTON UNIVERSITY
WASHINGTON, DC 20052

CHOI, WON S, MPH, PHD
PROFESSOR
DEPARTMENT OF POPULATION HEALTH
SCHOOL OF MEDICINE
UNIVERSITY OF KANSAS MEDICAL CENTER
KANSAS CITY, KS 66160

COHEN, DEBORAH A, MD, MPH
RESEARCH SCIENTIST III
DEPARTMENT OF RESEARCH AND EVALUATION
KAISER PERMANENTE SOUTHERN CALIFORNIA
PASADENA, CA 91101

DANILOVICH, MARGARET K, BA, DPT, PHD *
RESEARCH SCIENTIST AND DIRECTOR
LEONARD SCHANFIELD RESEARCH INSTITUTE
CJE SENIORLIFE
EVANSTON, IL 60201

DAVIS, RACHEL ELLEN, PHD, MPH, BA *
ASSOCIATE PROFESSOR
DEPARTMENT OF HEALTH PROMOTION,
EDUCATION, AND BEHAVIOR
ARNOLD SCHOOL OF PUBLIC HEALTH
UNIVERSITY OF SOUTH CAROLINA
COLUMBIA, SC 29208

FEDERMAN, ALEX D, MD, MPH
PROFESSOR
DEPARTMENT OF GENERAL INTERNAL MEDICINE
ICAHN SCHOOL OF MEDICINE AT MOUNT SINAI
NEW YORK, NY 10029

FREEDMAN, DARCY ANN, MPH, PHD
PROFESSOR AND DIRECTOR
SWETLAND CENTER FOR ENVIRONMENTAL HEALTH
DEPARTMENT OF POPULATION AND
QUANTITATIVE HEALTH SCIENCES
CASE WESTERN RESERVE UNIVERSITY SCHOOL OF
MEDICINE
CLEVELAND, OH 44106

GITTELSOHN, JOEL, PHD
PROFESSOR
DEPARTMENT OF INTERNATIONAL HEALTH
CENTER FOR HUMAN NUTRITION
BLOOMBERG SCHOOL OF PUBLIC HEALTH
JOHNS HOPKINS UNIVERSITY
BALTIMORE, MD 21205

HECKMAN, CAROLYN J, PHD
ASSOCIATE PROFESSOR
DEPARTMENT OF MEDICAL ONCOLOGY,
SECTION OF POPULATION SCIENCE
ROBERT WOOD JOHNSON MEDICAL SCHOOL
RUTGERS, THE STATE UNIVERSITY OF NEW JERSEY
NEW BRUNSWICK, NJ 08901

HERRING, SHARON J, BS, MD, MPH *
ASSOCIATE PROFESSOR
CENTER FOR OBESITY RESEARCH AND EDUCATION
LEWIS KATZ SCHOOL OF MEDICINE
TEMPLE UNIVERSITY
PHILADELPHIA, PA 19140

HUH, JIMI, BA, MA, PHD *
ASSOCIATE PROFESSOR
DEPARTMENT OF PREVENTIVE MEDICINE
KECK SCHOOL OF MEDICINE
UNIVERSITY OF SOUTHERN CALIFORNIA
LOS ANGELES, CA 90033

KOINIS MITCHELL, DAPHNE, PHD
PROFESSOR
DEPARTMENT OF PSYCHIATRY AND HUMAN BEHAVIOR AND
DEPARTMENT OF PEDIATRICS
BROWN MEDICAL SCHOOL
PROVIDENCE, RI 02903

LAUMBACH, ROBERT JOHN, MD, MPH *
ASSOCIATE PROFESSOR
DEPARTMENT OF ENVIRONMENTAL AND
OCCUPATIONAL HEALTH
RUTGERS SCHOOL OF PUBLIC HEALTH
PISCATAWAY, NJ 08854

LONG, KRISTIN ANN, AB, PHD *
ASSISTANT PROFESSOR
DEPARTMENT OF PSYCHOLOGICAL & BRAIN SCIENCES
BOSTON UNIVERSITY
BOSTON, MA 02215

MAIR, CHRISTINA FURBER, BS, MPH, PHD *
ASSISTANT PROFESSOR
DEPARTMENT OF BEHAVIORAL
AND COMMUNITY HEALTH SCIENCES
UNIVERSITY OF PITTSBURGH AT PITTSBURGH
PITTSBURGH, PA 15213-2535

MOON, RACHEL Y, MD *
PROFESSOR
DEPARTMENT OF PEDIATRICS
SCHOOL OF MEDICINE
UNIVERSITY OF VIRGINIA
CHARLOTTESVILLE, VA 22908

NELSON, TOBEN FREDRICK, BA, MS, SCD *
ASSOCIATE PROFESSOR
DIVISION OF EPIDEMIOLOGY
AND COMMUNITY HEALTH
SCHOOL OF PUBLIC HEALTH
UNIVERSITY OF MINNESOTA
MINNEAPOLIS, MN 55454

NICCOLAI, LINDA M, PHD
PROFESSOR
DEPARTMENT OF EPIDEMIOLOGY OF MICROBIAL DISEASES
YALE SCHOOL OF PUBLIC HEALTH
NEW HAVEN, CT 06520

PATEL, MINAL R, BA, MPH, PHD *
ASSOCIATE PROFESSOR
DEPARTMENT OF HEALTH BEHAVIOR & HEALTH
EDUCATION
SCHOOL OF PUBLIC HEALTH
UNIVERSITY OF MICHIGAN, ANN ARBOR
ANN ARBOR, MI 48109

SMITH, GREGORY C, PHD
PROFESSOR
HUMAN DEVELOPMENT CENTER
LIFESPAN DEVELOPMENT AND EDUCATIONAL SCIENCE
COLLEGE OF EDUCATION, HEALTH AND HUMAN SERVICES
KENT STATE UNIVERSITY
KENT, OH 44242

SORKIN, DARA H, PHD
ASSOCIATE PROFESSOR
DIVISION OF GENERAL INTERNAL MEDICINE
UNIVERSITY OF CALIFORNIA, IRVINE
IRVINE, CA 92697

STEVENS, ROBIN, AB, MPH, PHD *
ASSISTANT PROFESSOR
DEPARTMENT OF FAMILY AND COMMUNITY HEALTH
UNIVERSITY OF PENNSYLVANIA SCHOOL OF NURSING
PHILADELPHIA, PA 19151

STONER, SUSAN A, BA, PHD *
RESEARCH SCIENTIST
ALCOHOL AND DRUG ABUSE INSTITUTE
UNIVERSITY OF WASHINGTON
SEATTLE, WA 98105

WANG, MAY C, DRPH, MPH
PROFESSOR
DEPARTMENT OF COMMUNITY HEALTH SCIENCES
FIELDING SCHOOL OF PUBLIC HEALTH
UNIVERSITY OF CALIFORNIA LOS ANGELES
LOS ANGELES, CA 90095

WU, LI-TZY T, SCD
PROFESSOR
DEPARTMENT OF PSYCHIATRY
SCHOOL OF MEDICINE
DUKE UNIVERSITY MEDICAL CENTER
DURHAM, NC 27710

YIN, ZENONG, BED, MA, PHD *
PROFESSOR
DEPARTMENT OF KINESIOLOGY, HEALTH AND NUTRITION
COLLEGE OF EDUCATION AND HUMAN DEVELOPMENT
UNIVERSITY OF TEXAS AT SAN ANTONIO
SAN ANTONIO, TX 78249

YOUNG, HENRY N, PHD *
KROGER ASSOCIATE PROFESSOR
DEPARTMENT OF CLINICAL AND ADMINISTRATIVE PHARMACY
UNIVERSITY OF GEORGIA
ATHENS, GA 30602

ZHU, MOTAO, MD, MS, PHD *
PROFESSOR
DEPARTMENT OF PEDIATRICS
CENTER FOR INJURY RESEARCH AND POLICY
COLLEGE OF MEDICINE
OHIO STATE UNIVERSITY
COLUMBUS, OH 43205

MAIL REVIEWER(S)

ANWAR, MOHD, PHD
ASSOCIATE PROFESSOR OF COMPUTER SCIENCE
RTI SCHOLAR
CENTER FOR ADVANCED STUDIES IN IDENTITY SCIENCES
SECURE AND USABLE SOCIAL MEDIA AND NETWORKS LAB
NORTH CAROLINA A AND T STATE UNIVERSITY
GREENSBORO, NC 27411

WANG, RUI, BS, MS, PHD
ASSOCIATE PROFESSOR
DEPARTMENT OF POPULATION MEDICINE
HARVARD PILGRIM HEALTH CARE INSTITUTE
HARVARD MEDICAL SCHOOL
BOSTON 02115

SCIENTIFIC REVIEW OFFICER

WU, PING, PHD
SCIENTIFIC REVIEW OFFICER
CENTER FOR SCIENTIFIC REVIEW
NATIONAL INSTITUTES OF HEALTH
BETHESDA, MD 20892

EXTRAMURAL SUPPORT ASSISTANT

NJOKU, PHILIP C
EXTRAMURAL SUPPORT ASSISTANT
DIVISION OF AIDS, BEHAVORIAL, POPULATION SCIENCES
HEALTHCARE DELIVERY AND METHODOLOGIES (HDM)
NATIONAL INSTITUTES OF HEALTH
BETHESDA, MD 20892

* Temporary Member. For grant applications, temporary members may participate in the entire meeting or may review only selected applications as needed.

Consultants are required to absent themselves from the room during the review of any application if their presence would constitute or appear to constitute a conflict of interest.
